# Performance of four crop model for simulations of wheat phenology, leaf growth, biomass and yield across planting dates

**DOI:** 10.1371/journal.pone.0197546

**Published:** 2018-06-14

**Authors:** Jamshad Hussain, Tasneem Khaliq, Ashfaq Ahmad, Javed Akhtar

**Affiliations:** 1 Agro-climatology Laboratory, department of agronomy, university of agriculture, Faisalabad-Pakistan; 2 Institute of soil and environment sciences, University of Agriculture, Faisalabad-Pakistan; College of Agricultural Sciences, UNITED STATES

## Abstract

Robustness of four wheat simulation model were tested with 2-year field experiments of three cultivars across a wide range of sowing dates in two different climatic regions: Faisalabad (semi-arid) and Layyah (arid), in Punjab-Pakistan. Wheat growing season temperature ranged from -0.1°C to 43°C. The wide series of sowing dates was a unique opportunity to grow the wheat in an environment which temperatures varies from -0.1°C to 43°C. The CERES-Wheat, Nwheat, CROPSIM-Wheat and APSIM-Wheat model were calibrated against the least-stressed treatment for each wheat cultivar. Overall, the four models described performance of early, optimum and late sown wheat well, but poorly described yields of very late planting dates with associated high temperatures during grain filling. The poor accuracy of simulations of yield for extreme planting dates point to the need to improve the accuracy of model simulations at the high end of the growing temperature range, especially given the expected future increases in growing season temperature. Improvement in simulation of maximum leaf area index of wheat for all models is needed. APSIM-Wheat only poorly simulated days to maturity of very and extremely late sown wheat compared to other models. Overall, there is a need of improvement in function of models to response high temperature.

## Introduction

Hot and dry region are expected to be particularly vulnerable to climate change associated yield losses associated with increased temperature. Temperature and heat fluctuation negatively affects the morphological, physiological and yield contributing factors of crops. For example, an increase in temperature at flowering stage may cause pollen sterility in crops [1]. Increasing temperature affects wheat growth and development, resulting in smaller grains at 25–35°C due to shorter grain filling duration and reduced photosynthetic efficiencies at temperatures above 30°C. Asseng et al. [2] reported 6% reduction of global wheat yield with 1°C increase of temperature during the most comprehensive analysis to date using crop simulation models.

Crop models have a higher uncertainty in their simulation at elevated temperature due to their incorporated structures and functions [3]. Reducing the uncertainty surrounding the quantification of climate change impacts in models is a major concern of crop modelers [4–7]. For better simulation of crop responses under increasing temperature and CO_2,_ is also important for crop modelers to improve models for better use in changing temperature and heat stress. Though model results are more sensitive to stress during reproductive stages as compared to vegetative stages [8], simulations could use improvement to reflect heat stress at both stages [9]. Currently, most studies of the impacts of heat stress on crop growth are performed in growth chambers [10, 11], temperature gradient tunnels [12] or temperature free-air controlled enhancement (T-FACE) [13, 14] systems. Data of FACE, FATE, and glassware experiment are usually used for formulation of models used to quantify the climate change impacts [7]. These techniques may not accurately represent real responses in field crop production systems. The results of controlled environments cannot be extrapolated to natural field conditions owing to natural differences in solar radiation, wind and evaporation requirements [15]. Craufurd et al. [16] suggested that crop science experiments were urgently required to evaluate and improve crop models of heat stress that is likely under future climate impact projections. Similarly, Liu et al. [17] tested four different models in pot experiments (phytotrons) but suggested that models should be tested against field experiment results.

The primary objective of this study was to evaluate the four crop models (CERES-Wheat, Nwheat, CROPSIM-Wheat and APSIM-Wheat) with phenology, leaf area maximum, above ground biomass and yield of field grown wheat across early to extremely late planting time.

## Materials and methods

### Field experiments

Field trails were conducted at Agronomic Research Area, University of Agriculture, Faisalabad and Agronomic Research Station Karor, Layyah, during the years 2013–14 and 2014–15. The altitudes of Faisalabad and Layyah are 184 m and 143 m, respectively. The experiments were laid out following split plot arrangement keeping 11 planting dates (16^th^ October to 16^th^ March with interval of 15–16 days) in main plots and varieties (Galaxy-2013, Punjab-2011 and Lasani-2008) in subplots. Seed bed was well prepared to sow wheat using seed rate 100 kg ha^-1^ at 22 cm row to row distance. Nitrogen, phosphorus and potassium were applied were applied at the rate of 120, 85 and 60 kg ha^-1.^ Irrigation water was applied without giving water stress to crop. All other crop husbandry practices were kept same. Soil, crop management, crop phenology, leaf growth, yield, and weather data of both locations were collected following the standard procedures and methods.

### Model descriptions

Crop models are different in their structure, functions and parameter values [3]. Four crop models, DSSAT-CERES-Wheat, DSSAT-Nwheat, DSSAT-CROPSIM-Wheat and APSIM-Wheat were calibrated and evaluated against eleven planting dates at two different locations (Faisalabad and Layyah) during two growing seasons, 2013–14 and 2014–15.

#### DSSAT-CERES-Wheat

The DSSAT-CERES-Wheat model [18] under the shell of Decision Support System for Agro-technology Transfer (DSSAT v4.7) is the most cited wheat model; it has been tested and evaluated around the globe e.g., [19–23]. CERES-Wheat has been widely used for exploring agronomic options, breeding preferences, edaphic factors and climatic factors. This model has the capacity to simulate the developmental stages of wheat; growth of leaves, stem and grains; and biomass based on light interception and stresses.

#### APSIM-Wheat

The Agricultural Production Systems Simulator (APSIM) for wheat (v7.8) is an Australian based wheat model which has the ability to simulate the soil transformations such as nitrogen, water, crop residue, crop growth, development and their interactions [24]. APSIM-Wheat has been evaluated around the globe under different soils, climate, temperatures, CO_2_, planting dates, water, plant populations and cultivars [25–29]. APSIM-wheat simulates wheat growth on a daily time-step [30] by calculating thermal time from the difference between base temperature and 3-hourly crown temperatures derived from the daily maximum and minimum temperatures. The thermal time is then accumulated to determine the phonological development of the crop. The biomass accumulation is based on radiation use efficiency (RUE). Biomass partitioning rates to different plant parts vary with crop development stage and re-translocation begins at the stage of starting grain filling [28].

#### DSSAT-Nwheat

Recently, Nwheat has been embedded in DSSAT v4.7 as APSIM-Nwheat model. This model has been tested under the shell of APSIM-Nwheat in many environments for temperature, carbon dioxide, nitrogen and water transformation in soil [29–33]. Transpiration efficiency was increased directly 1 to 1.37 with doubling CO_2_ from 350 to 700 ppm. DSSAT-Nwheat uses the same input data set as CERES-Wheat but requires more cultivar coefficients are needed to calibrate. A heat stress function was introduced in Nwheat by Asseng et al. [4] based on CERES-Wheat model [34].

#### DSSAT-CROPSIM-Wheat

DSSAT-CROPSIM-wheat is an integrated model in DSSAT v4.7 that simulates wheat development, growth and morphological parameters based on single plant then converts into whole plant population. Phenological stages are calculated on the concept of “Biological Days” a time measure that equates to chronological days under optimum conditions. It mainly simulates the major phenological stages as given in Zadoks’ scale. Biomass is accumulated through intercepted radiation and distributed largely based on demand. Critical crop stresses are always considered during simulation of wheat under low or high temperature, which may cause plant death. Similarly low temperature at anthesis may cause sterility and reduction in final number of grains.

### Model input data

Models require input data that describe daily weather, cultivar growth and development characteristics, management events, and soil characteristics. The minimum weather data requirements are daily temperature (minimum and maximum), solar radiation, rainfall, and station information (longitude and latitude). The models use different genetic coefficients for a cultivar such as: vernalization requirement, photoperiod sensitivity, thermal time requirement, kernel number per biomass, kernel growth rate, maximum stem dry weight, and phyllochron interval. Vernalization and photoperiod affect phenology between emergence and floral initiation. Grain yield potential is controlled by a coefficient of kernel number per ear and maximum kernel growth rate. Leaf appearance is associated with degree day accumulation by the phyllochron parameter. Main soil inputs include initial soil water content, lower and drained upper limits, saturated water content, water drainage and runoff coefficients, rooting growth factors, first stage evaporation, and soil albedo. Crop management information incudes planting date and depth, plant population, fertilizer and irrigation application rates and dates, as well as measured or estimated initial soil water and nitrogen content [35].

### Model calibration and genetic coefficients

Calibration is the process of adjusting each model’s parameters to reflect local conditions. Four models were calibrated with the 15^th^ November planting during 2013–14 at Faisalabad. This is a necessary step to ensure models provide useful information about the system of interest. It is also necessary to obtain genetic coefficients to represent any new cultivars used in a given modeling study. All four models have different genetic coefficients, which were adjusted as described in Table 1. Some soil parameters were also adjusted in the process of model parameterization. Each of the four models for each cultivars was calibrated using data collected from the least-stressed planting date 15^th^ November, 2013–14 at Faisalabad Genetic coefficients for local cultivars were not available. So, crop specific parameters were estimated through iteration approach [36] and comparison of simulated and observed data. First, crop specific parameters regarding crop phenology were estimated then growth and yield related genetic coefficients were determined in all crop models (Table 1). The rest of the model parameters were taken from the original model documentations. Subsequently, calibrated models were applied to the remaining treatments of 2013–14 and 2014–15 at Faisalabad and Layyah. These genetic coefficients may be further used by environmentalist, crop breeders and geneticists for exploration of wheat cultivars under semiarid and arid environment of South Asia especially Pakistan.

**Table 1:** Genetic coefficients of wheat models CERES-Wheat, DSSAT-Nwheat, CROPSIM-Wheat and APSIM-Wheat for cultivars Lasani-2008, Punjab-2011 and Galaxy-2013.

Model’s Parameter		Cultivars coefficients		
CERES-Wheat		Lasasni-2008	Punjab-2011	Galxy-2013
P1V	Days, optimum vernalizing temperature, required for vernalization	19	20	21
P1D	Photoperiod response (% reduction in rate/10 h drop in pp)	86	88	88
P5	Grain filling (excluding lag) phase duration (^o^C.d)	690	670	710
G1	Kernel number per unit canopy weight at anthesis (#/g)	20	18	19
G2	Standard kernel size under optimum conditions (mg)	36	37	38
G3	Standard,non-stressed mature tiller wt (incl grain) (g dwt)	1	1	1
PHINT	Interval between successive leaf tip appearances (oC.d)	70	74	75
DSSAT-Nwheat				
VSEN	Sensitivity to vernalization	1.9	1.9	1.9
PPSEN	Sensitivity to photoperiod	2.5	2.5	2.5
P1	Thermal time from seedling emergence to end of juvenile phase	400	400	410
P5	Thermal time (start of grain filling to maturity) (oC)	700	660	660
ADLAI	threshold aeration deficit (AF2) affecting LAI	0.86	0.9	0.99
Grno	Kernel number per stem weight (kernel/g-stem)	24.5	24.5	28.5
MXFIL	Potential kernel growth rate [mg kernel-1 day-1]	1.5	1.5	1.5
STMMX	Potential final dry weight of a single tiller (g stem-1)	3	3	3
Phint	Phyllochron interval (°C-days/leaf appearance)	115	115	110
CROPSIM-Wheat				
Pn (p1-8)	Duration of phase n where n is phase number (PVoC.D)	(600, 72, 132, 190, 50, 25, 155, 465)	(590, 70, 130, 180, 50, 25, 140, 455)	(590, 70, 130, 180, 50, 25, 140, 450)
VREQ	Vernalization effect (fr)	3	3	3
PHINT	Interval between successive leaf appearances. (oC.d)	90	90	90
LAFV	Increase in potential area of leaves,vegetative phase (fr/leaf)	0.07	0.06	0.05
SHWTS	Standard,non-stressed shoot dry weight (incl.grain),maturity (g)	2.5	2.3	2.3
G#WTS	Standard grain number per unit canopy weight at anthesis (#/g)	21	21	18
GWTS	Standard grain size, optimum conditions, normal plant density (mg)	33	35	37
APSIM-Wheat				
potential_grain_ filling_rate	Potential daily grain filling rate (g/rgrain/day)	0.0189	0.0189	0.0189
grains_per_gram _stem	Kernel number per stem weight at the beginning of grain filling (g)	22.9	22.9	22.1
tt_end_of_juvenile	Thermal time needed from sowing to end of juvenile (°Cdays)	470	470	470
tt_floral_initiation	Thermal time from floral initiation to flowing (°Cdays)	250	250.9	250
tt_flowering	Thermal time needed in anthesis phase (°Cdays)	209	209	399
tt_start_grain_fill	Thermal time from start grain filling to maturity (°Cdays)	480	480	480
max_grain_size	Maximum grain size (g)	0.0614	0.0614	0.0627
vern_sens	Sensitivity to vernalisation	2.09	2.09	2.09
photop_sens	Sensitivity to photoperiod	3.24	3.24	3.24

### Model evaluation

To check the accuracy of the model simulations, models were evaluated with the data recorded during both seasons 2013–14 and 2014–15 at site of Faisalabad and Layyah except the 15^th^ November planting date used for calibration. The output of models were compared using statistical metrics normalized root mean square error (NRMSE). NRMSE evaluates the average relative deviation between observed and simulated values in percentage. The output variable, index of agreement (d), is a dimensionless and bounded measure originally provided by Willmott [37] and commonly used to compare the match of observed and simulated data [38–40].

Simulation performance was evaluated by calculating the statistical indices below,

$$○\;RMSE=={\left[\underset{i=1}{\sum }{\left(P_{i}-O_{i}\right)}^{2}/n\right]}^{0.5}$$ (1)

$$○\;d=1-[\Sigma_{i=1}{\left(P_{i}-O_{i}\right)}^{2}/\Sigma_{i=1}{\left(\left|P_{i}\right|+|O_{i}|\right)}^{2}]$$ (2)

where, Pi is simulated grain yield and Oi is observed grain yield.

○NRMSE=RMSE/MeanObservedGrainYield×100 (3)

## Results

### Models calibration

CROPSIM-Wheat, CERES-Wheat, Nwheat and APSIM-Wheat model were calibrated (Table 2) to further evaluation and improvement suggestions.

**Table 2:** Percentage differences (%) between observed and simulated data of four models CROPSIM-Wheat, CERES-Wheat, DSSAT-Nwheat and APSIM-Wheat for calibration of cultivars coefficients of Lasani-2008, Punjab-2011 and Galaxy-2013.

cv. Lasani-2008					
	CROPSIM-Wheat	CERES-Wheat	DSSAT-NWheat	APSIM-Wheat	Mean
Days to Anthesis	0.96	-3.85	-2.88	3.85	-0.48
Days to Maturity	1.37	-2.05	0.00	3.42	0.68
Grain Yield (kg ha^-1^)	-1.10	3.58	-0.13	-4.73	-0.60
Biological Yield (kg ha^-1^)	0.72	-3.75	-6.71	2.95	-1.70
Harvest Index (%)	-1.73	7.53	7.01	-7.92	1.22
Maximum LAI (m^2^/m^2^)	0.00	-5.88	-0.78	0.00	-1.67
Mean	-1.4	0.6	1.1	-0.2	0
cv. Punjab-2011					
Days to Anthesis	0.98	0.00	0.00	0.00	0.25
Days to Maturity	0.70	1.41	3.52	0.70	1.58
Grain Yield (kg ha^-1^)	-3.23	-2.42	-1.10	0.40	-1.59
Biological Yield (kg ha^-1^)	4.59	-1.31	-2.32	0.68	0.41
Harvest Index (%)	-8.15	-1.25	1.36	-0.29	-2.08
Maximum LAI (m^2^/m^2^)	14.00	2.00	2.00	0.00	4.50
Mean	1.5	-0.3	0.6	0.2	0.5
cv. Galaxy-2013					
Days to Anthesis	-2.91	0.00	-0.97	-0.97	-1.21
Days to Maturity	-0.70	2.10	2.10	1.40	1.22
Grain Yield (kg ha^-1^)	0.44	-1.28	3.33	-0.71	0.45
Biological Yield (kg ha^-1^)	-2.45	-2.89	0.65	1.02	-0.92
Harvest Index (%)	2.83	1.71	2.72	-1.74	1.38
Maximum LAI (m^2^/m^2^)	-5.88	3.92	-0.98	-0.39	-0.83
Mean	0	-0.7	-0.6	-0.4	-0.4

#### Overall behavior and performance of models

Percentage difference (PD) allows a relative ranking of the performance of the models during calibration for days to anthesis and maturity, biological and grain yield and LAI maximum variables are presented below for three genotypes i.e.

Lasani−2008=APSIM−Wheat(0.2%)<CERES−Wheat(0.6%)<Nwheat(1.1%)<CROPSIM−Wheat(1.14%);

Punjab−2011=CERES−Wheat(0.3%)<APSIM−Wheat(0.2%)<Nwheat(0.6%)<CROPSIM−Wheat(1.5%);

Galaxy−2013=CROPSIM−Wheat(0%)<APSIM−Wheat(0.4%)<Nwheat(0.6%)<CERES−Wheat(0.7%).

Overall, results showed that models are calibrated well which can be used for developing virtual agronomic and breeding options for wheat in climate warming scenario. Crop modelers can easily explore the different options for developing virtual cultivars which has high photosynthesis efficiency, heat resistance and higher grain filling rate to offset the climate warming impacts.

### Evaluations and validation

**Fig 1:**
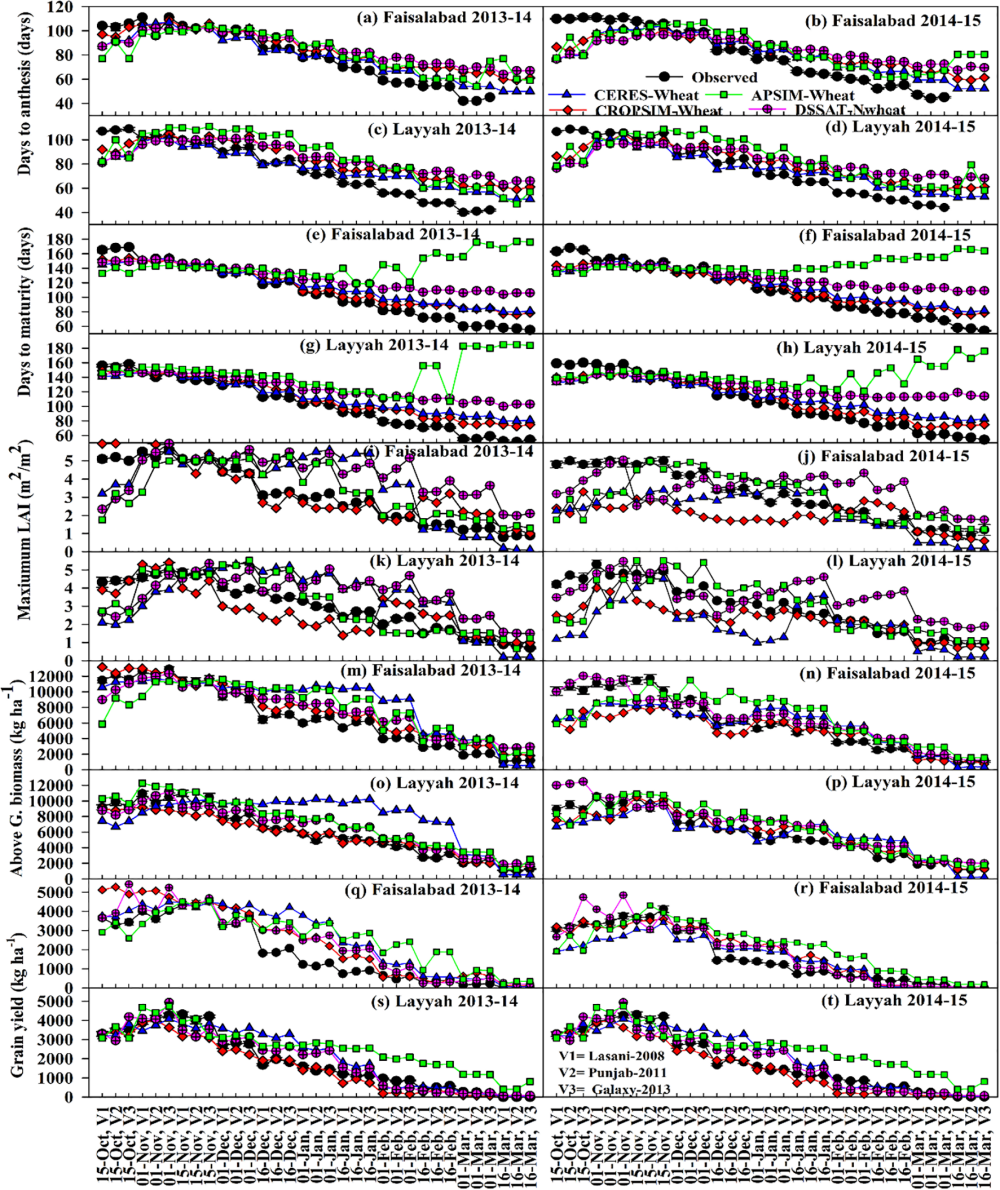
Evaluation of models CERES-Wheat, APSIM-Wheat, CROPSIM-Wheat and DSSAT-Nwheat with observed data for Days to anthesis (Fig a, b, c and d), Days to Maturity (Fig e, f, g and h), Maximum LAI (Fig i, j, k and l), Above ground biomass (Fig m, n, o and p) and grain yield (Fig q, r, s and t) of three varieties (Lasani-2008, Punjab-2011 and Galaxy-2013) during 11 planting dates 15^th^ October to 15^th^ march with interval of 15 days.

The performances of calibrated CROPSIM-Wheat, CERES-Wheat, Nwheat and APSIM-Wheat models were evaluated with independent data sets obtained from field-grown wheat during the 2013–14 and 2014–15 growing seasons at Faisalabad and Layyah for sowing dates from 15 October to 15 March except the one sowing dates which was used for calibration. Overall results with Normalized Root Mean Square Error (NRMSE) and index of agreement (d) presented in Tables 3 and 4 while performance on every sowing date of every model presented in Fig 1.

**Table 3:** Normalized root mean square error (%) of days to anthesis and maturity, grain and biological yield and leaf area index maximum of CROPSIM-Wheat, CERES-Wheat, DSSAT-Nwheat and APSIM-Wheat with observed data during 2013–14 and 2014–15 at Faisalabad.

		CROPSIM-Wheat		CERES-Wheat		Nwheat		APSIM-Wheat		Mean of Parameters	Means of Locations
		2013–14	2014–15	2013–14	2014–15	2013–14	2014–15	2013–14	2014–15		
Days To Anthesis	Faisalabad	17.3	29.6	12.3	25.9	22.2	34.6	21	35.8	24.8	34
Days To Maturity		12.0	11.6	13.9	14.3	25.9	25	26.9	50	22.5	
Grain yield (kg ha^-1^)		49.3	28.8	62.6	35.3	44.8	29.8	65.5	49.1	45.6	
Biological yield (kg ha^-1^)		16.9	38.5	42.1	35.3	24.2	20.3	39.2	40.8	32.2	
Lai maximum (m^2^/m^2^)		25.6	54.7	46.6	47.7	59.4	44.7	40.3	38.7	44.7	
Days to anthesis	Layyah	31.2	28.3	26	25.7	35.1	34.7	33.8	33.4	31.0	41.3
Days to maturity		12.5	11.1	16.3	16.7	29.8	29.6	59.6	53.7	28.7	
Grain yield (kg ha^-1^)		27.7	49.9	40.0	61.9	30.6	55.9	58.4	65.3	48.7	
Biological yield (kg ha^-1^)		16.6	18.1	72.5	25.3	21.1	26.8	27	24.1	28.9	
Lai maximum (m^2^/m^2^)		29	33.9	60.9	52	46.1	38.3	28.6	34.6	40.4	
Means		23.8	30.5	39.3	34	34	34	40	42.5	——	——
Mean of Models		27.1		36.7		34		41.9		——	——
Means of Years		34.27 (Season 2013–14)				35.25 (Season 2014–15)				——	——

**Table 4:** Index of agreement (d) of Days to anthesis and maturity, grain and biological yield and leaf area index maximum of CROPSIM-Wheat, CERES-Wheat, DSSAT-Nwheat and APSIM-Wheat with observed data during 2013–14 and 2014–15 at faisalabad.

		CROPSIM-Wheat		CERES-Wheat		DSSAT-Nwheat		APSIM-Wheat		Mean of Parameters	Means of Locations
		2013–14	2014–15	2013–14	2014–15	2013–14	2014–15	2013–14	2014–15		
Days To Anthesis	Faisalabad	0.88	0.74	0.95	0.82	0.78	0.55	0.81	0.59	0.77	0.81
Days To Maturity		0.96	0.96	0.93	0.94	0.76	0.69	0.36	0.19	0.72	
Grain yield (kg ha^-1^)		0.93	0.96	0.87	0.94	0.93	0.97	0.81	0.89	0.91	
Biological yield (kg ha^-1^)		0.98	0.87	0.86	0.89	0.94	0.97	0.84	0.87	0.90	
Lai maximum (m^2^/m^2^)		0.96	0.71	0.81	0.79	0.60	0.68	0.78	0.83	0.77	
Days to anthesis	Layyah	0.75	0.76	0.83	0.81	0.62	0.56	0.78	0.71	0.73	0.81
Days to maturity		0.96	0.97	0.92	0.92	0.68	0.57	0.29	0.27	0.70	
Grain yield (kg ha^-1^)		0.98	0.92	0.95	0.85	0.97	0.9	0.87	0.85	0.91	
Biological yield (kg ha^-1^)		0.97	0.97	0.63	0.93	0.96	0.94	0.94	0.95	0.91	
Lai maximum (m^2^/m^2^)		0.90	0.87	0.68	0.76	0.70	0.78	0.92	0.87	0.81	
Means		0.93	0.87	0.84	0.87	0.79	0.76	0.74	0.70	——	——
Mean of Models		0.90		0.85		0.78		0.72		——	——
Means of Years		0.83 (Season 2013–14)				0.80 (Season 2014–15)				——	——

#### Days to anthesis of different models

The values for NRMSE of CROPSIM-Wheat for days to anthesis (DTA) at Faisalabad during 2013–14 and 2014–15 were 17.3% and 29.6%, respectively. CERES-Wheat predictions had the highest (26%) and lowest NRMSE (12.3%) at Faisalabad during both season. Performance of N-wheat model was the best at Faisalabad during 2013–14 with NRMSE (34.6%) for DTA, though performance was similar for the remaining three datasets. The APSIM-Wheat model showed similar performance at all locations to Nwheat. Simulated data of CERES-Wheat at Faisalabad during 2013–14 were close to observed data with d-value (0.95), while Nwheat performance was the poorest, returning the lowest d-value (0.55) during 2014–15 at Layyah. CROPSIM-wheat and APSIM wheat were also reasonable with d values ranging 0.74–0.88 and 0.59–0.81 at both locations, respectively. Wheat models predicting DTA well for 1^st^, 15^th^ November and 1^st^ December planting dates, but agreement among model outputs and observed values gradually decreased with delay in planting dates. CERES-Wheat simulations of DTA were closer to observed than other models; Nwheat showed the greater deviation from observed DTA regardless of location and year. For early planting (15^th^ October), model simulations underestimated DTA but overestimated DTA for both years and locations. The closest agreement between model outputs and field date were found for 1^st^, 15^th^ November and 1^st^ December sowing dates, as compared to earlier and later sowing.

#### Days to maturity of different models

Among four models, simulations of CROPSIM-Wheat were the closest to the observed DTM with minimum NRMSE (11.1%) at Layyah during 2014–15 while APSIM-Wheat simulated the DTM with maximum NRMSE (59.6%). The d-index showed that CROPSIM-Wheat performed well followed by CERES-Wheat, Nwheat and APSIM-Wheat during both growing season at both locations. Performance of models was poor with delayed planting dates for all three cultivars. Models substantially under simulated DTM for the 15^th^ October planting date at both locations and during both growing season except at Layyah during 2013–14. Models over predicted DTM for 15^th^ December to 15^th^ March planting dates. APSIM-Wheat very poorly over predicted DTM values for planting dates after 15^th^ December; Nwheat only performed slightly better. Overall behavior of CROPSIM-Wheat and CERES-Wheat models was the same at Faisalabad across both 2013–14 and 2014–15. In general, DTM decreased for later planting dates in observed values and models simulations, with the exception of APSIM-Wheat, where DTM increased with delay in planting dates (after 1^st^ January at Faisalabad for both years and Layyah for 2013–14, but not until 16^th^ February at Layyah for 2014–15). Overall, models did not simulate DTM well for late planting dates, which were under high temperatures.

#### Leaf area index maximum of different models

NRMSE among all models, locations and growing seasons was minimum 25.6% at Faisalabad during 2013–14 from CROPSIM-Wheat while maximum (59.4%) was found for Nwheat at Faisalabad during 2013–2014. NRMSEs 29% and 33.9% at Layyah during 2013–14 and 201–15, respectively showed the best performance of CROPSIM-Wheat followed by APSIM-wheat at same locations. CERES-Wheat simulated LAIX better at Faisalabad (with NRMSEs 46.66% and 47.7%) than at Layyah (with N-RMSEs 60.9% and 52%) for 2013–14 and 2014–15, respectively. Minimum d-index (0.60) was found using Nwheat at Faisalabad in 2013–14 while maximum d-index (0.97) was found for CROPSIM-Wheat at Faisalabad in 2014–15. Model performance of models measured by d-index was better at Faisalabad than Layyah for both growing seasons. Models unanimously under simulated LAIX at both locations and seasons for planting dates of 15^th^ October. Models simulated LAIX well for 1^st^ and 15^th^ November sowing dates. For planting dates between 1^st^ December and 16^th^ February, model deviation from observed was increased relative to earlier planting dates. The final two planting of 1^st^ and 16^th^ March showed improved model performance. CROPSIM-Wheat was the best model for LAIX in all planting dates, locations, and years. APSIM-Wheat over simulated LAIX for late planting dates and showed more variation among three varieties as compared to other models. APSIM-Wheat simulated all planting dates at Faisalabad and Layyah during both growing season 20134–14 and 2014–15 well. Like other models, APSIM-Wheat under estimated LAIX at 16^th^ October and 1^st^ November plantings, but simulated the other planting dates’ LAIX with less deviation from observed values. CERES-Wheat over simulated the LAIX from 15^th^ November to 1^st^ February planting dates for both 2013–14 and 2014–15, but under simulated the LAIX at 15^th^ October, 16^th^ February, 1^st^ and 16^th^ March plantings. CERES-Wheat performed differently in the 2014–15 growing season at both locations. CERES-Wheat under simulated LAIX of October to December planting dates, but simulated LAIX of later planting dates from January onward with less variation (Fig 1).

#### Biological yield of different models

During comparison of models, the maximum NRMSE of biological yield was produced at Layyah during 2013–14 (72.5%) by CERES-Wheat and the minimum at the same location and growing season by CROPSIM-Wheat (16.6%). CROPSIM-Wheat performance was better at Layyah than Faisalabad during 2013–14 than 2014–15. Similarly, Nwheat and APSIM-Wheat simulations were closer to observed values at Layyah than Faisalabad during both growing seasons. CERES-Wheat showed maximum deviation from observed values, with an NRMSE at Layyah during 2013–14 of 72.5% and a minimum during 2014–15 at Faisalabad (35.3%). Maximum d-index was calculated in CROPSIM-Wheat at Layyah during 2014–15 and minimum d-index was observed in case of CERES-Wheat during 2013–14 at Faisalabad. Index of agreement was over 0.8 at both locations and years in all models except CERES-Wheat at Layyah during 2013–14 (0.62).

Biological yield decreased gradually with delay in planting in both locations and growing seasons for all three cultivars, as shown Fig 1. CROPSIM-Wheat and Nwheat were better at simulating the biological yield than APSIM-wheat and CERES-Wheat. During 2014–15 at Faisalabad, Nwheat simulating biological yield changes with planting date well compared to other three models. CROPSIM-Wheat, CERES-Wheat and APSIM-Wheat under predicted biological yield for 15^th^ October and 1^st^ November planting dates, performed well for 15^th^ November and 1^st^ December plantings, and slightly over simulated biological yield for the remaining planting dates. CROPSIM-Wheat, Nwheat and APSIM-Wheat showed little variation for all planting dates of all cultivars. CERES-Wheat showed more variation, under simulating yield for 15^th^ October and 1^st^ November planting dates, over simulating for later planting dates, and under simulating biological yield of last planting date at Layyah during 2013–14. Simulated and observed lines at Layyah during 2014–15 were somehow similar.

#### Grain yield of different models

Grain yield is the most important parameter to evaluate model performance. Minimum NRMSE (27.7%) was found for Layyah during 2013–14 while maximum NRMSE (65.5%) was found in APSIM-wheat during 2013–14 at Faisalabad. CERES-Wheat’s NRMSE ranged from 35.3% to 62.6%. Nwheat simulated grain yield well with NRMSE range of 29.6–55.9%. The highest d-index was found for CROPSIM-Wheat. The d-index ranged between 0.8 to 0.98 across models, locations and years. At higher grain yielding dates, model performance was better than lower yield planting dates. Overall model response was decreased with delay in planting date for all three cultivars and both growing seasons at both Faisalabad and Layyah, with the exception of a few planting dates simulated by CERES-Wheat at Layyah during 2013–14. At early planting of 15^th^ October, CERES-Wheat showed same response as observed, but CROPSIM-Wheat highly over simulated grain yield. APSIM-Wheat and Nwheat responded well for cultivar Lasani-2008 and Punjab-2011 but under simulated grain yield for Galaxy-2013. All models began to over simulate grain yield for planting dates of 16^th^ December, 1^st^ January, and 16^th^ January but simulations more closely matched observed values for later planting dates. APSIM-Wheat over simulated grain yield for planting dates after 1^st^ December at Layyah during 2013–14 and 2014–15. CROPSIM-Wheat simulated grain yield well for all planting dates and cultivars. CERES-Wheat under simulated grain yield for 1^st^ and 15^th^ November planting dates but over simulated the yields for plantings from 1^st^ December to 16^th^ February. CERES-Wheat performance improved for the final three planting dates at Layyah during 2013–14. For 2014–15 at Layyah, models did not model grain yield changes with planting date well. CROPSIM-Wheat over simulated the grain yield of 16^th^ December, 1^st^ and 16^th^ January and under simulated grain yields of the 16^th^ February and 1^st^ March plantings. Nwheat over simulated grain yield of Galaxy-2013 for the 15^th^ October planting. Nwheat over simulated grain yield of the 16^th^ December, 1^st^ and 16^th^ January plantings; the other plantings date grain yields were simulated well. CERES-Wheat response was haphazard: it under simulated for 16^th^ October, 15^th^ November, and 1^st^ December plantings and over simulated for 16^th^ December, 1^st^ and 16^th^ January, 1^st^ and 16^th^ February plantings. APSIM-Wheat highly over simulated the grain yield of all planting dates at Layyah during 2014–15 with the exception of the 15^th^ October planting.

## Discussion

Many studies evaluated and improved crop models under high temperatures e.g. [27, 41] in order to better simulate the results of climate change impacts on crops. Liu et al.[17] tested four models (DSSAT-CERES-Wheat, DSSAT, Nwheat, APSIM-Wheat, and Wheat-Grow) under heat stress conditions in phytotrons at grain filling and anthesis stages and highlighted the need for improving model simulation of grain yield and its components through field experimentation. Precise simulation of wheat development is the first step for accurate simulation of biological and grain yield, as well as their components [42]. Genetic characteristics, photoperiod and temperature are the main determinants of crop stages, but temperature is a major determinant of phenological stages [43]. Asseng et al.[2] also reported that the phenology of wheat is mainly regulated by temperature. Four models of wheat simulated development stages like anthesis and maturity with NRMSE ranging 12.3–35.8% and 11.1–59.6%, respectively across both locations and growing seasons. Simulated phenology of four models varied from observed due to different simulation function of four crop models. Large variation among the models because of different assumptions for parameter functions [9] like which cardinal temperature. CROPSIM-Wheat, CERES-Wheat, Nwheat, and APSIM-Wheat predicted days to anthesis closely. However, CROPSIM-Wheat, CERES-Wheat and Nwheat in DSSAT 4.7 [44], predicted the days to maturity similarly while APSIM-Wheat showed increased days to maturity with delayed planting due to its incorporated functions of photoperiod, cardinal temperature and low temperature sensitivity. APSIM-Wheat model empirically calculates of mean crown temperature to determining thermal time from daily maximum and minimum temperature, and calculates temperature stress by daily mean temperature [24].

A good model integrates all crop parameters and the effect of stresses on these parameters for final grain yield [9]. Liu et al. [17] also pointed to the need to improve the heat response of APSIM-Wheat. CROPSIM-Wheat performed comparatively better, providing good simulation of days to anthesis and maturity and total above ground biomass. CROPSIM-Wheat’s grain yield calculation method and cultivar coefficients also contributed to the good model performance. This model simulated the yield on the basis of tillering following 2.5 leaves at main stem, and grain numbers are determined by the function of the difference between the above ground biomass and at the end of anthesis stage and earlier stage [45]. Performance of CERES-Wheat was not as good at simulating phenology, but its NRMSE ranged from 35.30% to 62.60% across sites and years. Overall, CERES-Wheat over simulated grain yield; the model showed less sensitivity to increasing temperature after anthesis. CERES-Wheat simulation of days to anthesis and maturity did not show an effect of high temperature during grain filling stage on grain size and filling duration as in the field. Liu et al. [17] similarly reported that CERES-Wheat underestimates heat effects on grain filling duration. Models calculation of grain numbers at flowering stage and grain size as a function of grain growth rate and biomass partitioning at the reproductive stage [46] have been recommended for modification to better reflect heat stress effects [7, 47]

Among four models of our study, Nwheat best simulated biomass, with NRMSE ranging from 21.6%-26.8%, followed by CROPSIM-Wheat (16.6%-38.5%), APSIM-Wheat-Wheat 24.1–40.8% and CERES-Wheat (25.3%-72.50%). Nwheat biomass outputs were more sensitive to heat stress effects than CERES-Wheat and CROPSIM-Wheat [35]. APSIM-Wheat simulation performance was reliable but tended to overestimate biomass and its components like days to maturity and grain yield. In particular, later planting dates were associated with increased days to maturity, days to biomass accumulation, and consequently over simulation of biomass. A likelier driver of biomass overestimation is leaf area over simulation; days to maturity were over simulated in all environments but leaf area was over simulated in the same cases in which biomass was over simulated.

RMSE of all four models were averaged at two locations during two years to check the performance for simulation of days to anthesis (27.93%) and maturity (25.55%), grain yield (48.71%), biological yield (30.55%) and leaf area index (42.57%). As in other studies, we recommend the improvement of the models’ response function for simulation of grain yield at high temperature and under heat stress [17, 35]. Furthermore, we found room for improvement in simulations of leaf area index in the current models tested [21]. Comparison of mean NRMSE of all models, parameters, locations showed similar response in both experimental years 2013–14 (34.27%) and 2014–15 (35.25%). Models’ performance was better at Faisalabad (41.28%) than Layyah (33.96%). Mean NRMSE for all model parameters at both locations during year 2013–14 and 2014–15 showed that Nwheat (33.95%) performed better than CERES-Wheat (36.67%) and APSIM-Wheat (41.991%) due to better response to changing photoperiod, temperature and genetic coefficient.

Evaluation of models showed their level of reliability of simulation under different environments and temperature regimes such Faisalabad and Layyah as well as early and late sowing dates. Hussain et al. [48] reported in review that high temperature under climate change scenario would affect badly to wheat in semiarid and arid environment. These changing impacts of temperature could be offset through breeding and agronomic adaptations. Agronomic adaptations such as efficient irrigation, adjusting planting dates (16^th^ November ± 10 days) and increasing nitrogen application (10%), could enhance crop yield. Development of virtual cultivars through crop simulation modeling would be a good recommendation for breeder for breeding heat and temperature resistant cultivars.

In crux, multiple models performed well in early (16^th^ October), optimum (1^st^ and 16^th^ November) and late (1^st^ and 16^th^ December, 1^st^ January) sowing, but for very late planting dates (16^th^ January, 1^st^ and 16^th^ February, 1^st^ and 16^th^ March) under high temperature, models performance was poor. Performance of models during evaluation was sequenced as CROPSIM-Wheat > Nwheat > CERES-Wheat > APSIM-Wheat. Model performance was least accurate at simulating field data on leaf area index followed by grain yield. These data from later planting date field experiments are important for evaluating model performance at high temperature and can be used to further improve crop models in areas where heat stress is likely.
